# Isolation and characterization of bacteriophages from the human skin microbiome that infect *Staphylococcus epidermidis*

**DOI:** 10.1093/femsmc/xtab003

**Published:** 2021-03-30

**Authors:** Luca G Valente, Melissa Pitton, Monika Fürholz, Simone Oberhaensli, Rémy Bruggmann, Stephen L Leib, Stephan M Jakob, Grégory Resch, Yok-Ai Que, David R Cameron

**Affiliations:** Department of Intensive Care Medicine, Inselspital, Bern University Hospital, University of Bern, Bern, Switzerland; Institute for Infectious Diseases, University of Bern, Bern, Switzerland; Graduate School for Cellular and Biomedical Sciences (GCB), University of Bern, Bern, Switzerland; Department of Intensive Care Medicine, Inselspital, Bern University Hospital, University of Bern, Bern, Switzerland; Graduate School for Cellular and Biomedical Sciences (GCB), University of Bern, Bern, Switzerland; Department of Cardiology, Inselspital, Bern University Hospital, University of Bern, Bern, Switzerland; Interfaculty Bioinformatics Unit and SIB Swiss Institute of Bioinformatics, University of Bern, Bern, Switzerland; Interfaculty Bioinformatics Unit and SIB Swiss Institute of Bioinformatics, University of Bern, Bern, Switzerland; Institute for Infectious Diseases, University of Bern, Bern, Switzerland; Department of Intensive Care Medicine, Inselspital, Bern University Hospital, University of Bern, Bern, Switzerland; Department of Fundamental Microbiology, University of Lausanne, Lausanne, Switzerland; Department of Intensive Care Medicine, Inselspital, Bern University Hospital, University of Bern, Bern, Switzerland; Department of Intensive Care Medicine, Inselspital, Bern University Hospital, University of Bern, Bern, Switzerland

**Keywords:** phage therapy, biofilms, coagulase-negative staphylococci

## Abstract

Phage therapy might be a useful approach for the treatment of nosocomial infections; however, only few lytic phages suitable for this application are available for the opportunistic pathogen, *Staphylococcus epidermidis*. In the current study, we developed an efficient method to isolate bacteriophages present within the human skin microbiome, by using niche-specific *S. epidermidis* as the host for phage propagation. *Staphylococcus epidermidis* was identified on the forehead of 92% of human subjects tested. These isolates were then used to propagate phages present in the same skin sample. Plaques were observable on bacterial lawns in 46% of the cases where *S. epidermidis* was isolated. A total of eight phage genomes were genetically characterized, including the previously described phage 456. A total of six phage sequences were unique, and spanned each of the major staphylococcal phage families; Siphoviridae (*n* = 3), Podoviridae (*n* = 1) and Myoviridae (*n* = 2). One of the myoviruses (vB_SepM_BE06) was identified on the skin of three different humans. Comparative analysis identified novel genes including a putative N-acetylmuramoyl-L-alanine amidase gene. The host-range of each unique phage was characterized using a panel of diverse staphylococcal strains (*n* = 78). None of the newly isolated phages infected more than 52% of the *S. epidermidis* strains tested (*n* = 44), and non-*S. epidermidis* strains where rarely infected, highlighting the narrow host-range of the phages. One of the phages (vB_SepM_BE04) was capable of killing staphylococcal cells within biofilms formed on polyurethane catheters. Uncovering a richer diversity of available phages will likely improve our understanding of *S. epidermidis*-phage interactions, which will be important for future therapy.

## Teaser

Human skin is a useful source of bacterial viruses (phages) that have the capacity to lyse pathogenic bacteria and to destroy biofilms.

## INTRODUCTION

Antibiotic treatment failure is one of the biggest threats to human health. Progresses in modern medicine have contributed to the emergence of difficult-to-treat bacterial infections; overuse of antibiotics has expedited the spread of antibiotic resistance (Davies and Davies 2010), and the increased use of surgical procedures that require the implantation of foreign material has provided an ecological niche for bacteria to cause infection. In these cases, bacteria often exist as biofilms, encased within a protective extracellular matrix (Donlan 2001). There is an urgent need for alternative treatment strategies that can target antibiotic resistant bacterial strains and eradicate biofilms.

One such strategy is the use of bacterial viruses (bacteriophages), known as phage therapy (PT). The very nature of bacteriophages make them attractive for the treatment of infections caused by antibiotic-resistant, biofilm-producing bacteria. Phages target receptors that are distinct from traditional antibiotics, which suggests that the likelihood of phage cross resistance in antibacterial resistant strains is low (Loc-Carrillo and Abedon 2011). They can also code for enzymes with polysaccharide depolymerization activity that can degrade bacterial biofilms (Hughes, Sutherland and Jones 1998). Finally, they can be highly specific, often at the subspecies level, which may limit the potential for microbiome dysbiosis as off-target commensals should not be infected (Loc-Carrillo and Abedon 2011).

*Staphylococcus epidermidis* is a Gram-positive opportunistic pathogen that ubiquitously colonizes human skin (Kloos and Musselwhite 1975). In theory, *S. epidermidis* represents an ideal target for PT; it is a frequent cause of nosocomial infections due to its capacity to generate robust biofilms on indwelling foreign material, and it has a propensity to develop or acquire antibiotic resistance (Otto 2009; Becker, Heilmann and Peters 2014; Lee *et al*. 2018). In practice, however, little is known about the potential of PT for the treatment of *S. epidermidis* infections *in vivo*. PT for the infections caused by the closely related pathogen *Staphylococcus aureus* has been evaluated in animal models (Prazak *et al*. 2019, 2020) and in humans (Petrovic Fabijan *et al*. 2020), however no similar studies have been performed for *S. epidermidis*.

One of the major factors limiting the progress of PT for *S. epidermidis* is the lack of phages suitable for therapy. These phages should be obligatorily lytic toward the target organism, easy to propagate, stable and not carry virulence or antibiotic resistance genes (Loc-Carrillo and Abedon 2011). Over 200 staphylococcal phage genomes are publically available (Oliveira *et al*. 2019), however, to our current knowledge, less than 10% of these fit the suitability criteria for *S. epidermidis* (Melo *et al*. 2014a,b; Gutierrez *et al*. 2015; Cater *et al*. 2017). Indeed, isolating phages that infect *S. epidermidis* from typical sources including hospital sewage and wastewater has proven challenging (Melo *et al*. 2014a).

Naturally, the human body is covered in an abundance of both bacteria and phages (Foulongne *et al*. 2012; Wylie *et al*. 2014; Hannigan *et al*. 2015), suggesting the skin may be a good site for phage mining. Early attempts at searching for *S. epidermidis* phages in this setting however were unsuccessful (Gutierrez *et al*. 2010). More recently, using a single *S. epidermidis* laboratory strain as the host for phage infection, Aswani *et al*.(2011) found *S. epidermidis* phages in the anterior nares of a limited number of human subjects (5.5% of those tested).

*Staphylococcus epidermidis* has evolved many elegant strategies to overcome phage infection, which has likely limited the availability of phages for PT. For example, the widespread laboratory strain RP62A possesses a type III-A clustered regularly interspaced short palindromic repeats (CRISPR)-Cas system, a type I restriction modification system and a eukaryotic-like serine/threonine kinase (Stk2), each of which protect the bacteria from infection (Gill *et al*. 2005; Depardieu *et al*. 2016; Maniv *et al*. 2016). In order to uncover a greater diversity of *S. epidermidis* phages, selecting appropriate bacterial strains for propagation that are generally sensitive to phages is of importance (Hyman 2019).

In the current study, we screened the skin of healthy volunteers for lytic phages using human host-specific *S. epidermidis* as the chassis for propagation. Using this approach, we isolated novel phages spanning each of the known *S. epidermidis* phage families.

## MATERIALS AND METHODS

### Isolation of human host-specific *S. epidermidis*

All sampling from healthy volunteers was performed in accordance with Swiss ethics approval #2019–00769. Bacteria was recovered from the forehead using dry swabs (Invasive sterile collection swab, Sarstedt). Biomass on the swabs was immersed in Trypticase Soy Broth (TSB; BD, Franklin Lakes, New Jersey, USA), aliquots were spread onto mannitol salt agar (MSA) plates, and plates were incubated overnight at 37°C. Approximately 10 colonies that did not show evidence of mannitol fermentation were taken and patched onto fresh MSA plates. After 6–8 h of incubation at 37°C, species determination was performed using a Microflex LT Matrix-Assisted Laser Desorption/Ionization (MALDI) Biotyper (Bruker, Billerica, Massachusetts, United States). Staphylococcal isolates taken from the skin (commensals), as well as clinical isolates used in this study are listed in Table S1 (Supporting Information).

### Bacteriophage isolation, replication and propagation

Phages were isolated from the same site as *S. epidermidis* bacteria. The forehead, from eyebrow to hairline, was thoroughly swabbed with dry sterile tissue (Medicomp 5 cm x 5 cm, Hartman, Germany), the swabs were immersed in sodium-magnesium (SM) buffer (100 mM NaCl, 8 mM MgSO_4_, tris-HCl, pH 7.4; Bonilla and Barr 2018) and vortexed. Samples were filtered using 0.22-µm filters (Rotilabo-syringe filters, Carl Roth, Germany) and concentrated to a target volume (< 500 µL) using ultra-centrifugal filters (Amicon Ultra-1510000 MWCO, Merck, Kenilworth, New Jersey, United States; 4000 × *g*, 4°C). In order to propagate phages, an overnight culture of host-specific *S. epidermidis* was diluted 1:1000 in sterile TSB and incubated at 37°C with constant shaking (190 rpm),  until the culture reached an optical density (OD; 600 nm) of 0.2. Bacterial hosts for propagation where then infected with the phage solution at a ratio of 5:1 (bacteria: phage) and incubated at 37°C with shaking for 4 h to allow for adsorption. The phage–bacteria suspension was centrifuged (3000 × *g*, 5–10 min, 4°C) in order to pellet bacteria and the supernatant was passed through a 0.22-μm filter. For phage detection, the double-layer agar method was implemented (Kropinski *et al*. 2009). The solution containing presumable phages was mixed with approximately 5×10^7^ colony forming units (CFU)/mL host-specific *S. epidermidis* in TSB containing 0.75% agar and 2 mM CaCl_2_ (‘soft TSA+CaCl_2_’), then poured on preset TSB agar (TSA) plates producing a uniform top layer. Plates were incubated at 37°C overnight in static conditions. After incubation, the establishment of cleared zones (plaques) in the bacterial lawn indicated the presence of viable phages. Individual plaques were picked and propagated in double layer agar plates at least two times in order to purify the phages. The final phage suspensions were generated by producing confluent lysis of host *S. epidermidis* lawns in double layer agar, extracting the phages in SM buffer, then passing the suspensions through 0.22 μm filters (Bonilla and Barr 2018).

### Genome sequencing and analysis

Phage total DNA was extracted as described previously (Jakociune and Moodley 2018). Phage lysates were centrifuged (161 400 × *g*, 2 h, 4°C) in a Centrikon T-1170 ultracentrifuge using a TST 41.14 rotor (Kontron Instruments, U.K.), then phage containing pellets were resuspended in SM buffer. In order to remove residual DNA and RNA, lysates were incubated with DNase I (6 U/mL) and RNase A (0.02 mg/mL) for 1.5 h at 37°C. Enzymes were then deactivated via the addition of 0.02 M ethylenediaminetetraacetic acid (EDTA). In order to digest the phage protein capsid, Proteinase K (0.05 mg/mL) was added and samples were incubated at 56°C for 1.5 h. Phage DNA was then purified using DNeasy Blood & Tissue Kits (Qiagen, Hilden, Germany).

For genome sequencing, libraries were generated using Nextera DNA flex Library Prep Kits (Illumina, San Diego, California, United States) and then sequenced using an Illumina MiSeq (2×150bp). Raw reads were first trimmed and filtered using fastp (Chen *et al*. 2018), then complete phage genomes were assembled using SPAdes (v3.14.1) in ––isolate mode (Bankevich *et al*. 2012). Genomes were annotated using a combination of prokka (Seemann 2014) and PHASTER (Arndt *et al*. 2016). Whole-phage genome comparisons were performed using BLASTn (Altschul *et al*. 1990). Circular representations of phage genomes were created using GCview (Grant and Stothard 2008). Protein sequence alignments and comparisons were generated using Clustal Omega and BLASTp (Altschul *et al*. 1997; Sievers *et al*. 2011). Phage 456, which was used in a previous study to prevent *S. epidermidis* biofilm formation on catheters *in vitro* (Curtin and Donlan 2006), was included in this study as a control, and was also genome sequenced. Genome-based phylogeny was inferred using VICTOR (Meier-Kolthoff and Goker 2017). Phage genomes were screened for virulence genes using VirulenceFinder 2.0 (Joensen *et al*. 2014) and for antibiotic resistance genes using the Comprehensive Antibiotic Resistance Database (CARD; Alcock *et al*. 2020). Assembled and annotated phage genome sequences were deposited at DDBJ/ENA/GenBank under the accession numbers listed in Table 2.

### Host range characterization

The host range of select phages was determined using the double layer agar ‘spot test’ method (Kropinski *et al*. 2009). *Staphylococcus epidermidis* strain F12 (Gutierrez *et al*. 2010) was used as the initial bacterial host for propagation of each phage in order to calculate and compare efficiencies of plating (E.O.P.), except for vB_SepP_BE03, which did not infect F12, and was instead propagated in SKNA73. The concentration of each phage was normalized to ∼10^9^ plaque forming units (PFU)/mL. The infectivity of each phage, defined by the propensity to produce quantifiable plaques, was determined using a collection of 78 staphylococcal strains (Table S1, Supporting Information).

Briefly, overnight cultures of bacteria were mixed with soft TSA+CaCl_2_ to a concentration of ∼5×10^7 ^colony forming units (CFU)/mL, and then poured onto preset TSA plates. The top layer containing bacteria was allowed to dry, phages were serially diluted, and 4 µL of each dilution was plated onto the surface of the agar. Plates were incubated overnight at 37°C. The limit for detection was 2.5×10^2^ PFU/mL.

### Determination of phage capacity for lysogeny

Genetic analysis revealed the presence of integrase genes for vB_SepS_BE01 and vB_SepS_BE02. During the host range characterization of vB_SepS_BE01, putative lysogens were detected for three of the 78 bacterial hosts tested (SKN21, SKNA21 and SKNA60), as revealed by bacterial growth in the centre of the phage lysis zone. To determine if vB_SepS_BE01 was temperate, putative lysogens of *S. epidermidis* strains SKN21, SKNA21 and SKNA60 were tested for the presence of integrated vB_SepS_BE01 by performing polymerase chain reactions (PCR). Genomic DNA was extracted from bacteria using DNeasy Blood & Tissue Kits (Qiagen) according to the protocol of the manufacturer. PCR was performed using primers specific to amplify a 962 bp fragment of vB_SepS_BE01 (5’-GGCGTCGTTATGGTTAATGG-3’ and 5’-GGTCTTGTTGTTCGGATTGC-3’). For vB_SepS_BE02, no putative lysogens were identified during the host range characterization. Instead, to determine if vB_SepS_BE02 was temperate and integrated into its host *S. epidermidis* strain (SKNA40), genomic DNA from SKNA40 was subjected to PCR using primers specific to amplify a 937 bp fragment of vB_SepS_BE02 (5’–CTCTATCGACCCTGTTAAGTGG-3’ and 5’-GGAAGAACACCATCAATGCC-3’). DNA products were analyzed using agarose gel electrophoresis.

### One-step growth curve determination

One-step growth experiments were performed as described previously (Cater *et al*. 2017). Each experiments were carried out in biological duplicate. The latent period was defined as the time period from adsorption to the beginning of the phage burst. The burst size was calculated as PFU/mL after burst divided by PFU/mL before burst. For the siphoviruses vB_SepS_BE01 and vB_SepS_BE02, the media was supplemented with 10 mM CaCl_2_, as described previously (Lu *et al*. 2019).

### Determination of bacteriophage-insensitive mutant (BIM) frequency

BIM frequencies were determined as previously described (O'Flynn *et al*. 2004). Briefly, an overnight culture of *S. epidermidis* strain F12 was mixed with phages and plated in soft TSA+CaCl_2_ using the double-layer agar method. Plates were incubated overnight at 37°C. All bacterial colonies (BIMs) formed were counted and the BIM frequency was calculated by dividing the surviving CFU by the initial CFU. All experiments were performed in triplicate.

### Biofilm disruption assays–96-well plate method

Biofilms were produced in 96-well microtitre plates and quantified essentially as described elsewhere (O'Toole 2011). Briefly, overnight cultures of bacteria were diluted 1:100 in TSB supplemented with 0.25% glucose (‘TSB+glucose’, 150 µL per well). Plates were incubated statically at 37°C for 24 h. Following incubation, planktonic cells were removed by inverting the plates, and then biofilms were washed by submerging the plates in sterile water. Approximately 1×10^8 ^PFU of phages diluted in TSB were added (200 µL per well) and the plates were incubated for 4 h at 37°C, as described elsewhere (Gutierrez *et al*. 2015). SM-buffer was used for the untreated control. Following treatment, biofilms were washed three times then stained with crystal violet (CV; 0.06%) for 15 min at room temperature (RT). Excess CV was removed, and plates were again submerged three times in water. Biofilms were dried for 1 h at RT. Finally, acetic acid (30%) was added to each well and OD was measured at 550 nm (MiniMax 300 Imaging Cytometer, Molecular Devices, San Jose, California, United States). A total of five wells were used per condition, and each experiment was performed in biological triplicate. Comparisons between untreated and phage treated biofilms were determined using Kruskal–Wallis tests with multiple comparisons corrected for using Dunn's multiple comparisons test. Data were considered significantly different when *P* ≤ 0.05.

### Biofilm disruption assays—polyurethane catheter method

Staphylococcal biofilms were generated on 1.5 cm sections of polyurethane catheter in 12-well plates. Catheter sections were submerged in TSB+glucose containing ∼10^7^ CFU/mL of bacteria then plates were incubated at 37°C for 24 h. Following incubation, catheter sections were rinsed three times in PBS (pH 7.4) and transferred to a well of a new 12-well plate containing 2 mL of either NaCl 0.9% (untreated control), vancomycin (5 mg/mL) or vB_SepM_BE04 (1×10^9^ PFU/mL) then incubated for 4 h at 37°C. Each catheter section was rinsed three times in phosphate buffered saline (PBS, pH 7.4) then transferred to a 1.5 mL microcentrifuge tube containing 1 mL of PBS. Biofilm detachment was achieved via three cycles of vortexing (90 s) and sonicating (10 min), and surviving CFU were enumerated on TSA. A total of six catheter sections were analysed per condition. Statistical comparisons were performed using two-way ANOVA and pairwise differences were determined using Tukey's multiple comparisons test. Data were considered significantly different when *P* ≤ 0.05.

## RESULTS

### Efficient method for isolation of *S. epidermidis* phages

**Figure 1.**
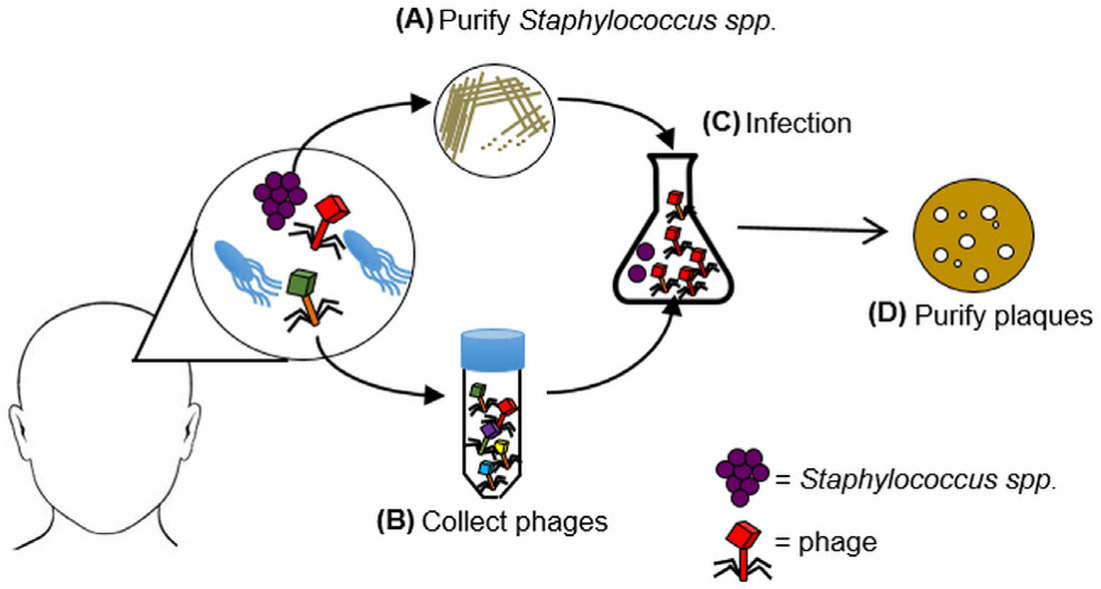
Schematic representation of the phage isolation method used in this study. Samples were taken from the skin of healthy volunteers. Commensal staphylococci were isolated using selective media then the species of each bacteria was determined via matrix-assisted laser desorption/ionization time-of-flight mass spectrometry (MALDI-TOF MS) **(A)**. From the same skin site, a second sample was taken, filtered and phages were concentrated by centrifugation **(B)**. *S. epidermidis* was then used as the host for phage propagation **(C)**. Phage plaques were produced in double-layer agar plates for further purification and characterization **(D)**.

We reasoned that lytic *S. epidermidis* phages would be present in niches where *S. epidermidis* is abundant. We targeted the forehead of healthy volunteers; *S. epidermidis* is frequently found on the head (Kloos and Musselwhite 1975) and it gives a large surface area that could be screened in a non-invasive fashion. Each individual's specific *S. epidermidis* strain was then used as the host for phage propagation and subsequent isolation (Fig. 1). A total of 26 individuals from central Switzerland were screened using this approach (Table 1). A total of 24 harbored *S. epidermidis* on the forehead (92.3%), which validated the use of this sampling site. Phage containing plaques were identified on lawns of human-host specific bacteria for 11 of the 24 subjects where *S. epidermidis* was isolated (46%). In one instance, multiple plaques with distinct morphologies were observed and propagated for further characterization. In the 13 cases where plaques were not identified, at least one additional *S. epidermidis* isolate (up to 4) from the same individual's skin was tested for plaque formation, however, no plaques were observed.

**Figure 2.**
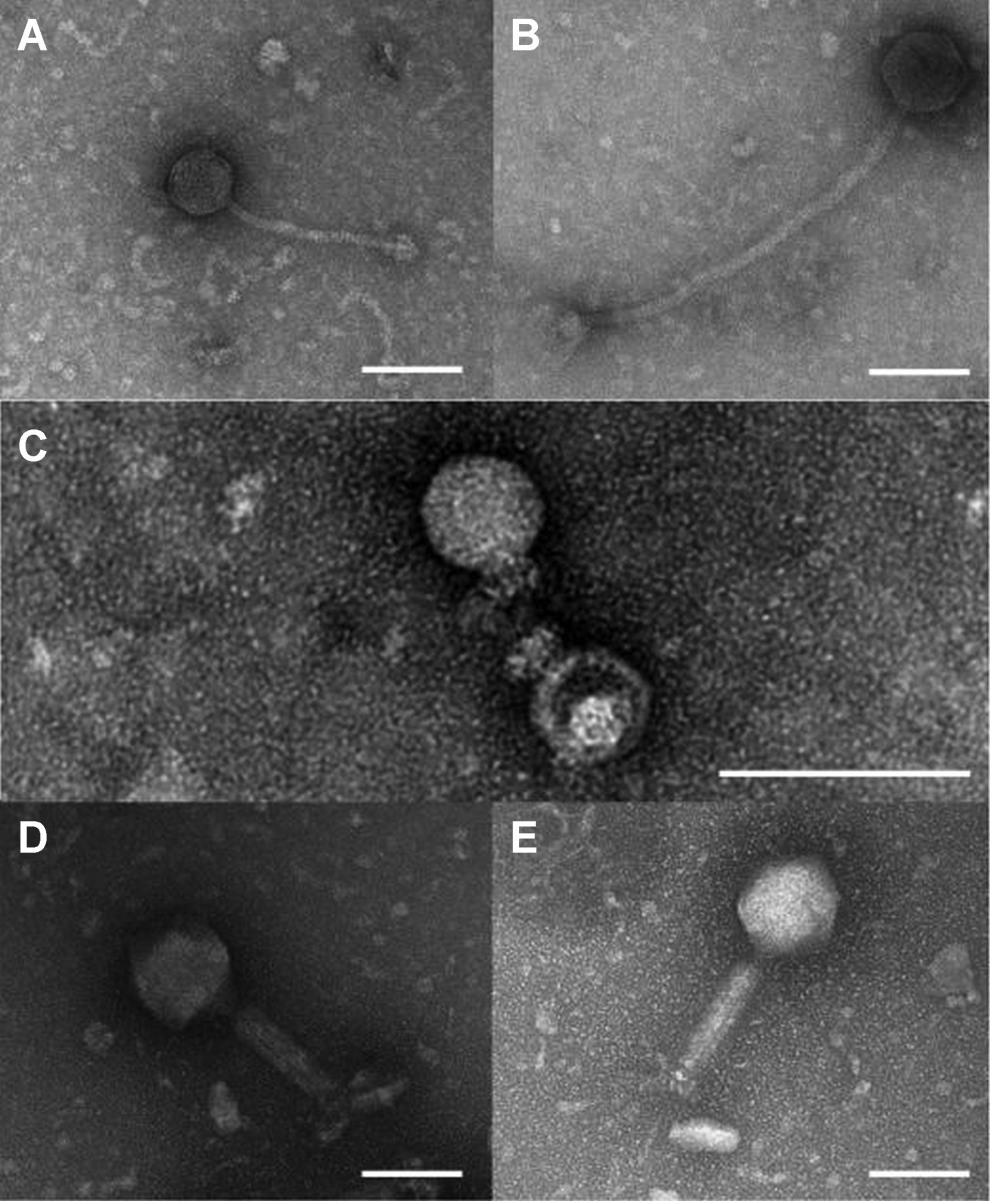
Electron micrographs of phages that infect *S. epidermidis*. Newly identified phages spanned the three major staphylococal phage families; Siphoviridae (vB_SepS_BE01 [**A**], vB_SepS_BE02 [**B**]), Podoviridae (vB_SepP_BE03 [**C**]) and Myoviridae (vB_SepM_BE04 [**D**], vB_SepM_BE06 [**E**]). White lines indicate 100 nm.

**Figure 3.**
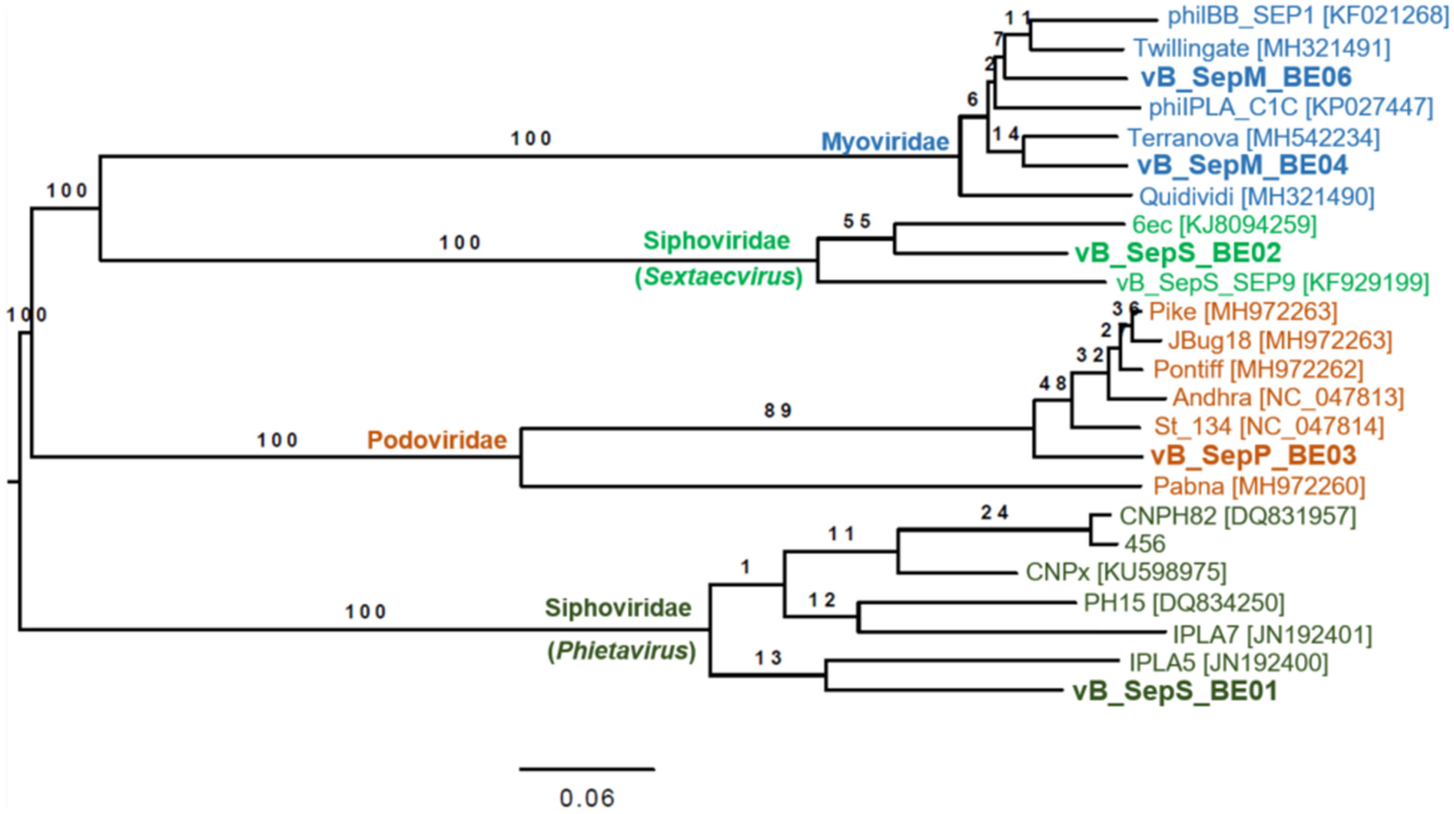
Phylogenetic relatedness of bacteriophages infecting *S. epidermidis*. Genome-BLAST Distance Phylogeny (GBDP) was determined using VICTOR using the D6 formula (Meier-Kolthoff and Goker 2017). Numbers above each branch are GBDP pseudo-bootstrap support values inferred from 100 replicates. All publically available *S. epidermidis* phage sequences were included. Phages identified in the current study are represented using bold font.

**Table 1.** Presence/absence of *S. epidermidis* and bacteriophages on the skin of healthy volunteers.

Subject	*S. epidermidis*	Plaques	Phage name
1	+	−	
2	+	+	vB_SepM_BE07
3	+	−	
4	+	+	vB_SepM_BE06
5	+	−	
6	−	−	
7	−	−	
8	+	+	vB_SepS_BE01, vB_SepM_BE09
9	+	+	#
10	+	+	vB_SepS_BE02
11	+	−	
12	+	−	
13	+	−	
14	+	−	
15	+	+	vB_SepM_BE04
16	+	+	#
17	+	−	
18	+	−	
19	+	−	
20	+	+	#
21	+	+	#
22	+	−	
23	+	−	
24	+	−	
25	+	+	vB_SepP_BE03
26	+	+	#

# putative phages were not stably propagated.

In total, seven phages that could be stably propagated and maintained at high concentration (>10^9^ PFU/mL) were selected for genetic and functional characterization.

### Human skin harbors diverse *S. epidermidis* phages

Exploiting the human skin microbiome to propagate specific phages allowed us to isolate at least one phage from each of the major staphylococcal phage families; Siphoviridae, Podoviridae and Myoviridae (Table 2, Figs 2 and 3).

**Table 2.** Genome characteristics of phages used in this study.

Name	Family	Genus	Lifestyle	Length (bp)	%GC	CDS	Reference	Accession
vB_SepS_BE01	Siphoviridae	*Phietavirus*	Temperate	42 718	34.8	70	This study	MT596498
456	Siphoviridae	*Phietavirus*	Temperate	43 393	34.7	73	Curtin and Donlan (2006)	MT596497
vB_SepS_BE02	Siphoviridae	*Sextaecvirus*	Virulent	95 233	29.4	141	This study	MT596499
vB_SepP_BE03	Podoviridae	Unclassified Picovirinae	Virulent	18 271	30	20	This study	MT596500
vB_SepM_BE04	Myoviridae	*Sepunavirus*	Virulent	142 331	27.9	208	This study	MT596501
vB_SepM_BE06	Myoviridae	*Sepunavirus*	Virulent	140 659	28	200	This study	MT596503
vB_SepM_BE07	Myoviridae	*Sepunavirus*	Virulent	140 661	28	200	This study	MT596504
vB_SepM_BE09	Myoviridae	*Sepunavirus*	Virulent	140 668	28	201	This study	MT596506
vB_SepM_phiIPLA-C1C	Myoviridae	*Sepunavirus*	Virulent	140 961	28	203	Gutierrez *et al*. (2015)	See ref.

**Figure 4.**
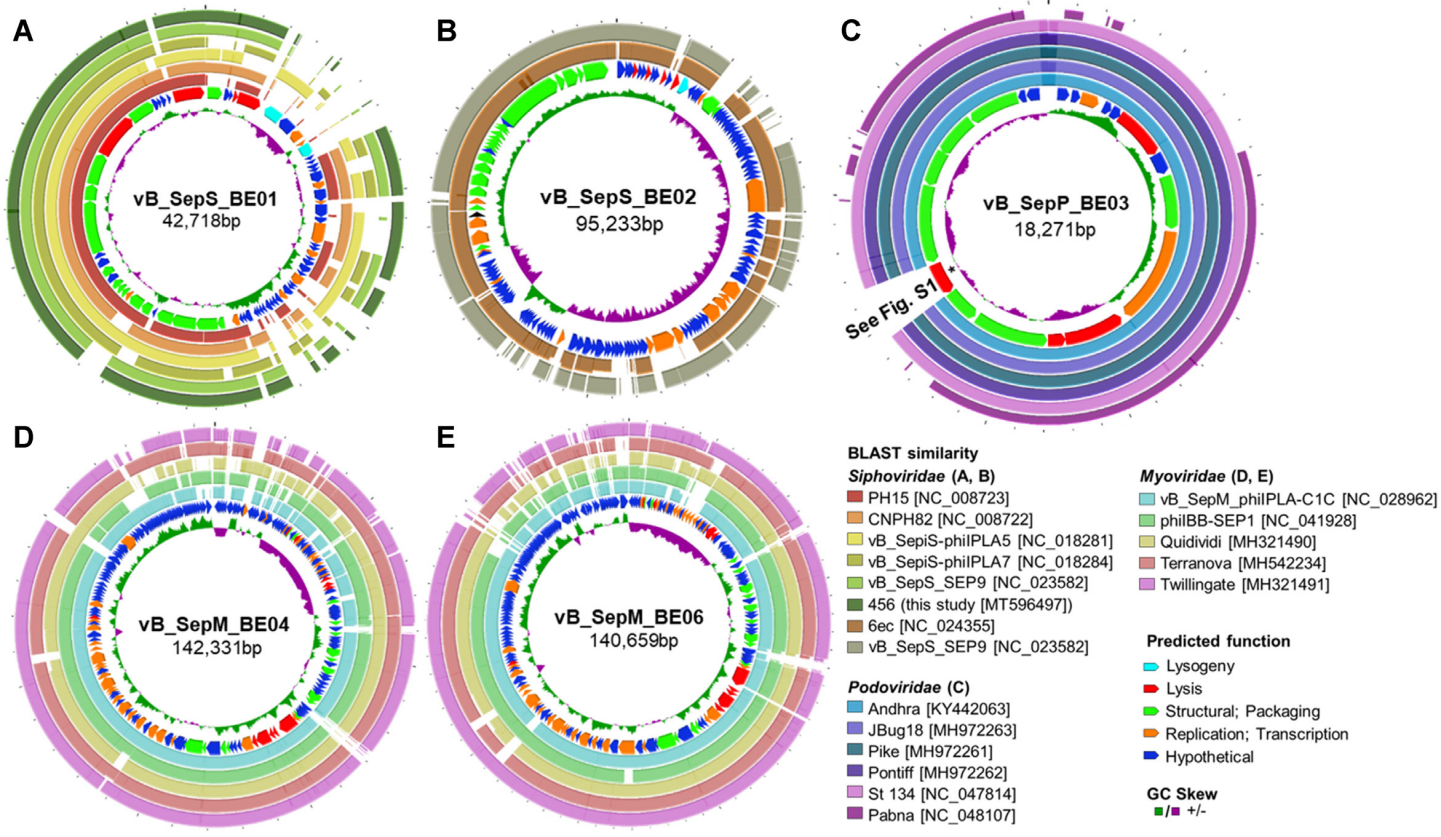
Circular genome representation of five novel bacteriophages isolated from human skin. For each panel, the inner ring illustrates the GC skew. The second ring shows putative coding sequences; the color of each arrow indicates predicted function. The remaining outer rings illustrate BLASTn sequence similarities for comparisons with other staphylococcal bacteriophages. vB_SepS_BE01 shows sequence similarity with phages of the *Phietavirus* genus **(A)**, and vB_SepS_BE02 is similar to phages of the *Sextaecvirus* genus **(B)**. vB_SepP_BE03 is a podovirus **(C)**. An asterisk is included to highlight a sequence predicted to code for an N-acetylmuramoyl-L-alanine amidase (see Figure S1, Supporting Information). The final two phages, vB_SepM_BE04 **(D)** and vB_SepM_BE06 **(E)**, are similar to known myoviruses of the *Sepunavirus* genus. Plots were generated using the CGView server (Grant and Stothard 2008).

Of the siphoviruses, vB_SepS_BE01 belongs to the *Phietavirus* genus, possessing a genome of 42 718 base pairs (bp) that is homologous to previously described *S. epidermidis* phages vB_SepS-phiIPLA5 (Identity 96%; Query coverage 80%), vB_SepS-phiIPLA7 (Identity 97%; Query coverage 65%) and CNPx (Identity 97%; Query coverage 69%; Fig. 4A; Gutierrez *et al*. 2012; Depardieu *et al*. 2016). Its genome is organized in six functional modules typical of the *Siphoviridae* family (lysogeny, DNA replication, packaging, head, tail and lysis) (Xia and Wolz 2014). This genetic arrangement indicated that vB_SepS_BE01 is a temperate phage. Indeed, vB_SepS_BE01 produced lysogens when exposed to three independent *S. epidermidis* isolates (SKN21, SKNA21, SKNA60; Table S1, Supporting Information). vB_SepS_BE01 integration was confirmed by targeted PCR using purified genomic DNA from the lysogens of each *S. epidermidis* strain. We also sequenced phage 456, which has been used previously to disrupt *S. epidermidis* biofilms (Curtin and Donlan 2006). Phage 456 also belongs to the *Phietavirus* genus, has a genome length of 43 393 bp,  is very similar to phage CNPH82 (99.7% Identity; 98% Query coverage) and is predicted to be lysogenic based on the presence of putative genes coding for an integrase, cl-like and cro-like repressors and an anti-repressor.

The second siphovirus isolated in the current study was vB_SepS_BE02, which belongs to the *Sextaecvirus* genus and has a genome length of 95 233 bp. It is homologous to *S. epidermidis* phages vB_SepS_SEP9 (98% Identity; 74% Query coverage) and 6ec (99% Identity; 82% Query coverage; Fig. 4B; Aswani *et al*. 2014; Melo *et al*. 2014a). As described for other *Sextaecviruses*, vB_SepS_BE02 possesses an integrase, but does not have the complete set of genes that are predicted to be required for lysogeny (Aswani *et al*. 2014; Melo *et al*. 2014a). Unlike vB_SepS_BE01, exposing 44 diverse *S. epidermidis* strains to vB_SepS_BE02 in double-layer agar plates did not result in the production of any detectable lysogens. Additionally, the phage was not integrated into the genome of the *S. epidermidis* strain used to identify and propagate the phage (SKNA40; Table S1, Supporting Information), as determined by PCR.

vB_SepP_BE03 is a podovirus with a genome 18 271 bp in length that shares high sequence homology with previously described staphylococcal phages Pike (95% Identity; 95% Query coverage), JBug18 (94% Identity; 95% Query coverage), St134 (96% Identity; 95% Query coverage) Andhra (93% Identity; 95% Query coverage) and Pontiff (94% Identity; 95% Query coverage; Fig. 4C; Cater *et al*. 2017; Culbertson *et al*. 2019). vB_SepP_BE03 has a unique gene, absent from all other podoviruses that infect *S. epidermidis* (Fig. 4). This gene is predicted to code for a putative N-acetylmuramoyl-L-alanine amidase as determined by ClustalW alignment and conserved domain analysis (pfam01510; Amidase_2 domain and cd06583; peptidoglycan recognition protein domain; Figure S1, Supporting Information).

The remaining six phages identified in this study belonged to the *Myoviridae* family. A total of two unique genome sequences were determined; vB_SepM_BE04 (Fig. 4D) and vB_SepM_BE06 (Fig. 4E and Table 2). vB_SepM_BE04 is 142 331 bp in length and shares homology with phiIBB-SEP1 (97% Identity; 89% Query coverage). vB_SepM_BE06 is 140 659 bp in length and is also homologous to Terranova (98% Identity; 89% Query coverage). Surprisingly, the remaining two phages sequenced (vB_SepM_BE07 and vB_SepM_BE09) were almost identical to vB_SepM_BE06 (>99% identity, >99% coverage), even though they each were isolated from the skin of different human subjects. Given the high degree of similarity between vB_SepM_BE06, vB_SepM_BE07 and vB_SepM_BE09, only vB_SepM_BE06 was selected for further functional characterization.

None of the phages harbored known or putative antibiotic resistance genes or virulence genes as determined by VirulenceFinder 2.0 and the CARD, respectively.

### *Staphylococcus epidermidis* phages can infect diverse staphylococcal isolates including methicillin-resistant examples

**Figure 5.**
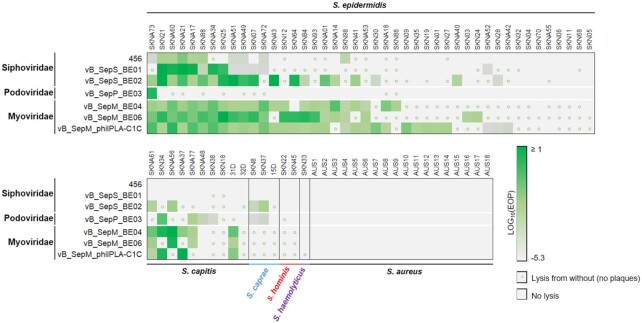
Host-range characterization of staphylococci and staphylococcal phages. Seven unique phages were tested for their capacity to infect 72 diverse staphylococcal isolates. Efficiency of plating (E.O.P.) was determined by comparing plaque forming units (PFU)/mL from each interaction with those determined for the strain used for phage propagation (*S. epidermidis* F12). Lysis from without, whereby faint inhibition halos were observed without countable plaques was indicated by small circles.

One-step growth curves were generated (Figure S2, Supporting Information), and the latency period, and burst size was calculated for each phage (Table S2, Supporting Information). The infectivity profile of each of the unique phages identified in the study, as well as control phages vB_SepM_phiIPLA-C1C and 456 identified elsewhere (Curtin and Donlan 2006; Gutierrez *et al*. 2015), was assessed against a panel of 78 different staphylococcal strains (Table S1, Supporting Information), resulting in a matrix of more than 500 bacteria–phage interactions (Fig. 5). The strain panel consisted of *S. epidermidis* (*n* = 44; 21 methicillin-resistant *S. epidermidis* [MRSE], 23 methicillin-sensitive *S. epidermidis* [MSSE]; 28 isolates of clinical origin, 16 commensals), *S. aureus* (*n* = 18; all methicillin-resistant *S. aureus* [MRSA]), *S. capitis*, (*n* = 10), *S. caprae* (*n* = 3), *S. hominis* (*n* = 2), *S. haemolyticus* (*n* = 1). Interactions were determined using ‘spot-tests’. When countable plaques were observed, a multiplicity of infection was calculated compared to the control *S. epidermidis* strain F12. When a lysis halo was observable on the surface of agar in undiluted phage samples, but no countable plaques were formed, this was considered ‘lysis from without’ (Abedon 2011).

Focusing on *S. epidermidis*, the panel of seven phages infected 2–70% of isolates (median, 45%). A total of 14 out of 21 MRSE isolates tested were susceptible to at least one phage. Eight of the 44 (17.8%) isolates tested were not susceptible to any of the phages. The control myovirus, vB_SepM_phiIPLA-C1C isolated by Guiterrez *et al*. (2015) infected the most *S. epidermidis* isolates (70%). The two myoviruses identified in this study infected 50–52% of the *S. epidermidis* isolates tested. The sole podovirus (vB_SepP_BE03) infected only one of the *S. epidermidis* tested. Of the siphoviruses, the virulent *Sextaecvirus* vB_SepS_BE02 infected 45% of the *S. epidermidis*, whereas the temperate phages of the *Phietavirus* genus (vB_SepS_BE01 and 456) had the narrowest host ranges (23–27%).

None of the phages infected any of the MRSA isolates tested, and additional members of the coagulase negative staphylococci (CoNS) were only infected infrequently (Fig. 5). The frequency of bacteriophage-insensitive mutants was determined and the results are presented in Table S2 (Supporting Information).

### vB_SepM_BE04 is active against staphylococcal biofilms

The capacity of the newly isolated phages to remove biofilms was first tested using standard high-throughput biofilm assays in 96-well plates. Each phage was tested against three different CoNS isolates; a methicillin-resistant *S. epidermidis* (MRSE, SKN25), a methicillin-sensitive *S. epidermidis* (MSSE, SKNA73) and an *S. capitis* (SKN34; Figure S3, Supporting Information). The myovirus vB_SepM_BE04 removed biofilm biomass most effectively in the high-throughput assay, and it was selected for further characterization.

**Figure 6.**
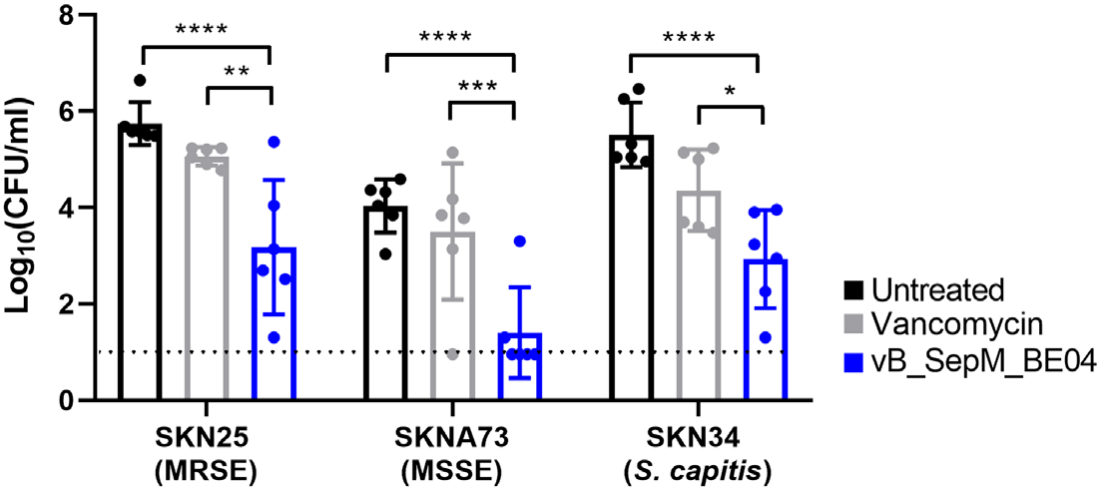
Treatment of staphylococcal biofilms established on catheter sections. Biofilms were grown on 1.5 cm polyurethane catheters for 24 h *in vitro* then treated with either vancomycin (5 mg/mL) or vB_SepM_BE04 (∼10^9^ plaque forming units/ml). Viable colony forming units (CFU) where determined on agar plates following treatment. Data were compared using two-way ANOVA (treatment effect *P* < 0.0001; strain effect *P*< 0.0001; interaction *P* = 0.896). Simple pairwise comparisons between treatments are shown, and significant differences are illustrated using asterisks (*P < 0.05i>; **P< 0.01i>; ***P< 0.001; ^****^P< 0.0001). The black dotted line represents the limit of detection (1 log_10_CFU/mL). MRSE, methicillin-resistant *S. epidermidis*; MSSE, methicillin-sensitive *S. epidermidis*.

The ability of vB_SepM_BE04 to kill cells within biofilms formed on polyurethane catheter sections was determined, and compared to the standard-of-care for methicillin-resistant staphylococci, vancomycin 5 mg/mL (Mermel *et al*. 2009), for each of the three CoNS isolates (Fig. 6). Significant differences between viable CFU were observed across strains and treatments (*P* < 0.0001 for each), and there was no interaction between factors (*P* = 0.896, two-way ANOVA). When observing the main effects of treatment, vancomycin produced a modest reduction in CFU when compared to the untreated control (≤1 log_10_CFU/mL, *P* = 0.035, Tukey's multiple comparisons test). vB_SepM_BE04 treatment resulted in a significant reduction of viable CFU when compared to both untreated (1.4–2.4 log_10_CFU/mL, *P*< 0.0001), and vancomycin treated catheters (1.4–2.1 log_10_CFU/mL *P*< 0.0001).

## DISCUSSION

Phage mining expeditions typically target an untreated water source, often relying on one, or a limited number of bacterial host-strains for propagation. With few exceptions, this approach has proven challenging for the isolation of phages that infect *S. epidermidis* (Gutierrez *et al*. 2015; Culbertson *et al*. 2019). Recent advances in microbiome sequencing and analysis has helped to illustrate the rich diversity of phages present in natural environments, many of which appear similar to known staphylococcal phages (Hannigan *et al*. 2017; van Zyl *et al*. 2018). Thus, it is a paradox that so few phages specific for *S. epidermidis* are available for therapeutic application. In the current study, we tried to maximize the likelihood of identifying cultivable phages from human skin by providing a familiar bacterial host taken from the same niche as the driver of phage propagation (Fig. 1). At least one unique representative from each of the major staphylococcal phage families (siphoviridae, podoviridae and myoviridae) was isolated and functionally characterized.

Siphoviridae are often temperate, and considered inappropriate for therapy. Temperate phages can integrate foreign genetic material into the genome of the host strain, which may inadvertently equip the infective bacteria with genes that contribute to antibiotic resistance or enhanced pathogenicity (Loc-Carrillo and Abedon 2011). Indeed vB_SepS_BE01 was shown to lysogenize three distinct *S. epidermidis* strains. In contrast, vB_SepS_BE02 did not have the typical set of genes required by siphoviruses to undergo lysogeny, and we did not observe any putative lysogens. This suggests that vB_SepS_BE02 may be only able to undergo a lytic cycle and is thus a virulent phage, similar to what has been proposed for the other known *S. epidermidis* phages from the *Sextaecvirus* genus (Aswani *et al*. 2014; Melo *et al*. 2014a).

A single podovirus was identified (vB_SepP_BE03, Fig. 4C). Podoviruses are attractive candidates for therapy due to their obligatorily lytic nature and compact genomes (Culbertson *et al*. 2019). Most of the vB_SepP_BE03 genome could be functionally annotated (13/20 putative genes), which is desirable when considering therapeutic applications. However, vB_SepP_BE03 displayed a very narrow host range, which may not be desirable for future therapy.

The remaining phages were lytic myoviruses. In contrast to vB_SepP_BE03, most genes coded for by the myoviruses belonged to the so-called ‘viral dark matter’—which includes the large number of phage genes with unknown function (Roux *et al*. 2015). It was somewhat surprising that highly similar myoviruses where isolated from the skin of three different human subjects. These observations however, support a growing body of evidence that has identified identical and near identical phages in seemingly diverse sampling subjects including geographically distinct aquatic environments (Mizuno *et al*. 2013; Kalatzis *et al*. 2017; van Zyl *et al*. 2018). In the context of *S. epidermidis*, the phages identified from the skin have a narrow host range (Fig. 5); however, their primary host is abundant in the location from which we sampled. This may result in low selective pressure, genetic conservation and phage persistence, which is in agreement with the interpretations of other research groups (Hannigan *et al*. 2017; van Zyl *et al*. 2018).

In addition to isolating intact phages that could be useful for therapy, comparative genomic analyses performed in this study also identified novel phage genes, which may help to improve our understanding of *S. epidermidis*–phage interactions. For example, vB_SepP_BE03 coded for a unique putative N-acetylmuramoyl-L-alanine amidase. In place of this gene, other podoviruses that infect *S. epidermidis* code for a different amidase (Andrah_gp14) with low amino acid similarity (28% identity; 40% query coverage). Andrah_pg14 is thought to be a tail-associated amidase with moderate hydrolytic activity, which functions in the early stages of infection (Cater *et al*. 2017). Given the sequence differences between Andrah_pg14 and the amidase from vB_SepP_BE03 (BESEP3_00014), it is plausible that they also function differently, which may influence infectivity. Additionally, vB_SepM_BE04, which outperformed standard-of-care antibiotics for the treatment of biofilms formed on catheters *in vitro*, may code for an as yet unidentified biofilm degrading enzyme. Identifying new phage proteins that are involved in the infection process and/or the degradation of biofilm paves the way for the synthetic reprogramming of phages with tailored functionality, as has been performed for phages that infect other pathogens (Dunne *et al*. 2019).

Therapeutic phage products fit within two paradigmatic categories; *sur*-*mesure*—for a specific application, or *prêt-à-porter*—‘one-size-fits-all’ (Pirnay *et al*. 2011). A *prêt-à-porter* approach requires a product that can infect the majority of clinical isolates of the target pathogen. For *S. aureus*, we have used a four-phage cocktail effective against >90% of isolates (Prazak *et al*. 2019, 2020). This approach is not feasible for *S. epidermidis* phages identified in the current study, which infected only ∼50% of strains tested (Fig. 5). We propose a *sur-mesure* approach for *S. epidermidis* infections, whereby a patient's infective isolate is used to propagate phages from within the individual's own skin microbiome, producing a unique and targeted therapeutic.

In summary, we have developed a method for isolating diverse phages from the skin, using *S. epidermidis* as the target opportunistic bacterial pathogen. The results serve as a proof-of-concept for subsequent studies designed to broaden the arsenal of cultivable phages available for therapy.

## Supplementary Material

xtab003_Supplemental_Files
