# Supplementary material for: Isolation and characterization of bacteriophages from the human skin microbiome that infect Staphylococcus epidermidis
Source: FEMS Microbes. 2021 Mar 30;2:xtab003. doi: 10.1093/femsmc/xtab003 (PMC10117716; doi:10.1093/femsmc/xtab003)
Supplement: xtab003_Supplemental_Files [file xtab003_supplemental_files.zip › ValentePitton_supplemental_clean.docx]

**SUPPLEMENTAL MATERIAL**

**Isolation and characterization of bacteriophages from the human skin microbiome that infect *Staphylococcus epidermidis***

Luca Valente^1,2,3*^, Melissa Pitton^1,3*^, Monika Fürholz^4^, Simone Oberhaensli^5^, Rémy Bruggmann^5^, Stephen L. Leib^2^, Stephan M. Jakob^1^, Grégory Resch^6^, Yok-Ai Que^1^ and David R. Cameron^1,#^

1. Department of Intensive Care Medicine, Inselspital, Bern University Hospital, University of Bern, Bern, Switzerland

2. Institute for Infectious Diseases, University of Bern, Bern, Switzerland

3. Graduate School for Cellular and Biomedical Sciences (GCB), University of Bern, Bern, Switzerland

4. Department of Cardiology, Inselspital, Bern University Hospital, University of Bern, Bern, Switzerland

5. Interfaculty Bioinformatics Unit and SIB Swiss Institute of Bioinformatics, University of Bern, Bern, Switzerland

6. Department of Fundamental Microbiology, University of Lausanne, Lausanne, Switzerland

**Running title:** Therapeutic phages from human skin

***Joint first-authors.** Order was determined by coin-toss.

**#Corresponding author.**

David R. Cameron, PhD
Department of Intensive Care Medicine
Inselspital; Bern University Hospital
3010 Bern, Switzerland

Tel: +41 31 632 42 55; E-Mail: davidrobert.cameron@insel.ch

**Table S1.** Staphylococcal strains used in this study.

| **Species** | **Strain ID** | **Source** | **Location** | **MRS^a^** | **Reference** |
| --- | --- | --- | --- | --- | --- |
| *S. epidermidis* | SKN01 | Clinical | Bern, Switzerland | Yes | This study |
|  | SKN03 | Clinical | Bern, Switzerland | Yes | This study |
|  | SKN04 | Clinical | Bern, Switzerland | Yes | This study |
|  | SKN05 | Clinical | Bern, Switzerland | Yes | This study |
|  | SKN06 | Clinical | Bern, Switzerland | Yes | This study |
|  | SKN09 | Clinical | Bern, Switzerland | Yes | This study |
|  | SKN11 | Clinical | Bern, Switzerland | Yes | This study |
|  | SKN19 | Clinical | Bern, Switzerland | Yes | This study |
|  | SKN21 | Clinical | Bern, Switzerland | Yes | This study |
|  | SKN24 | Clinical | Bern, Switzerland | Yes | This study |
|  | SKN25 | Clinical | Bern, Switzerland | Yes | This study |
|  | SKN27 | Clinical | Bern, Switzerland | Yes | This study |
|  | SKN28 | Clinical | Bern, Switzerland | Yes | This study |
|  | SKN30 | Clinical | Bern, Switzerland | Yes | This study |
|  | SKN32 | Clinical | Bern, Switzerland | Yes | This study |
|  | SKN35 | Clinical | Bern, Switzerland | Yes | This study |
|  | SKN41 | Clinical | Bern, Switzerland | Yes | This study |
|  | SKN43 | Clinical | Bern, Switzerland | Yes | This study |
|  | SKN64 | Clinical | Lausanne, Switzerland | Yes | This study |
|  | SKN68 | Clinical | Lausanne, Switzerland | Yes | This study |
|  | SKN70 | Clinical | Lausanne, Switzerland | Yes | This study |
|  | SKN07 | Clinical | Bern, Switzerland | No | This study |
|  | SKN12 | Clinical | Bern, Switzerland | No | This study |
|  | SKN84 | Clinical | Lausanne, Switzerland | No | This study |
|  | SKN86 | Clinical | Lausanne, Switzerland | No | This study |
|  | SKN88 | Clinical | Lausanne, Switzerland | No | This study |
|  | SKN93 | Clinical | Lausanne, Switzerland | No | This study |
|  | SKN98 | Clinical | Lausanne, Switzerland | No | This study |
|  | SKNA49 | Commensal | Bern, Switzerland | No | This study |
|  | SKNA51 | Commensal | Bern, Switzerland | No | This study |
|  | SKNA01 | Commensal | Bern, Switzerland | No | This study |
|  | SKNA14 | Commensal | Bern, Switzerland | No | This study |
|  | SKNA17 | Commensal | Bern, Switzerland | No | This study |
|  | SKNA18 | Commensal | Bern, Switzerland | No | This study |
|  | SKNA21 | Commensal | Bern, Switzerland | No | This study |
|  | SKNA33 | Commensal | Bern, Switzerland | No | This study |
|  | SKNA34 | Commensal | Bern, Switzerland | No | This study |
|  | SKNA40 | Commensal | Bern, Switzerland | No | This study |
|  | SKNA42 | Commensal | Bern, Switzerland | No | This study |
|  | SKNA52 | Commensal | Bern, Switzerland | No | This study |
|  | SKNA55 | Commensal | Bern, Switzerland | No | This study |
|  | SKNA60 | Commensal | Bern, Switzerland | No | This study |
|  | SKNA72 | Commensal | Bern, Switzerland | No | This study |
|  | SKNA73 | Commensal | Bern, Switzerland | No | This study |
|  | F12 | unknown | unknown | unknown | (1) |
| *S. aureus* | AUS1 | Clinical | Bern, Switzerland | Yes | This study |
|  | AUS2 | Clinical | Bern, Switzerland | Yes | This study |
|  | AUS3 | Clinical | Bern, Switzerland | Yes | This study |
|  | AUS4 | Clinical | Bern, Switzerland | Yes | This study |
|  | AUS5 | Clinical | Bern, Switzerland | Yes | This study |
|  | AUS6 | Clinical | Bern, Switzerland | Yes | This study |
|  | AUS7 | Clinical | Bern, Switzerland | Yes | This study |
|  | AUS8 | Clinical | Bern, Switzerland | Yes | This study |
|  | AUS9 | Clinical | Bern, Switzerland | Yes | This study |
|  | AUS10 | Clinical | Bern, Switzerland | Yes | This study |
|  | AUS11 | Clinical | Bern, Switzerland | Yes | This study |
|  | AUS12 | Clinical | Bern, Switzerland | Yes | This study |
|  | AUS13 | Clinical | Bern, Switzerland | Yes | This study |
|  | AUS14 | Clinical | Bern, Switzerland | Yes | This study |
|  | AUS15 | Clinical | Bern, Switzerland | Yes | This study |
|  | AUS16 | Clinical | Bern, Switzerland | Yes | This study |
|  | AUS17 | Clinical | Bern, Switzerland | Yes | This study |
|  | AUS18 | Clinical | Bern, Switzerland | Yes | This study |
| *S. capitis* | SKN34 | Clinical | Bern, Switzerland | No | This study |
|  | SKN18 | Clinical | Bern, Switzerland | No | This study |
|  | SKN38 | Clinical | Bern, Switzerland | No | This study |
|  | SKNA48 | Commensal | Bern, Switzerland | unknown | This study |
|  | SKNA37 | Commensal | Bern, Switzerland | unknown | This study |
| *S. caprae* | SKN37 | Clinical | Bern, Switzerland | No | This study |
|  | SKN8 | Clinical | Bern, Switzerland | No | This study |
| *S. hominis* | SKN22 | Clinical | Bern, Switzerland | Yes | This study |
|  | SKN45 | Clinical | Bern, Switzerland | No | This study |
| *S. haemolyticus* | SKN33 | Clinical | Bern, Switzerland | No | This study |

^a^MRS, Methicillin-Resistant *Staphylococcus*

**References**

1. Gutierrez D, Vandenheuvel D, Martinez B, Rodriguez A, Lavigne R, Garcia P. 2015. Two Phages, phiIPLA-RODI and phiIPLA-C1C, Lyse Mono- and Dual-Species Staphylococcal Biofilms. Appl Environ Microbiol 81:3336-48.

**Table S2.** One-step growth curve characteristics and bacteriophage insensitive mutant (BIM) frequency

| **Name** | **Latency phase (min)** | **Burst size** | **BIM frequency^1^ (mean ± SD)** |
| --- | --- | --- | --- |
| vB_SepS_BE01 | 30 | 49.3 | N/A |
| vB_SepS_BE02 | 40 | 34.4 | 7.48x10^-6^ ± 1.08x10^-8^ |
| vB_SepP_BE03 | 30 | 25 | N/A |
| vB_SepM_BE04 | 25 | 16.4 | 1.41x10^-5^ ± 2.96x10^-6^ |
| vB_SepM_BE06 | 40 | 9 | 1.38x10^-5^ ± 8.72x10^-7^ |

**^1^**BIM frequencies were not determined for vB_SepS_BE01 or vB_SepP_BE03 as they did not cause complete lysis of the host strain in double layer agar.

**
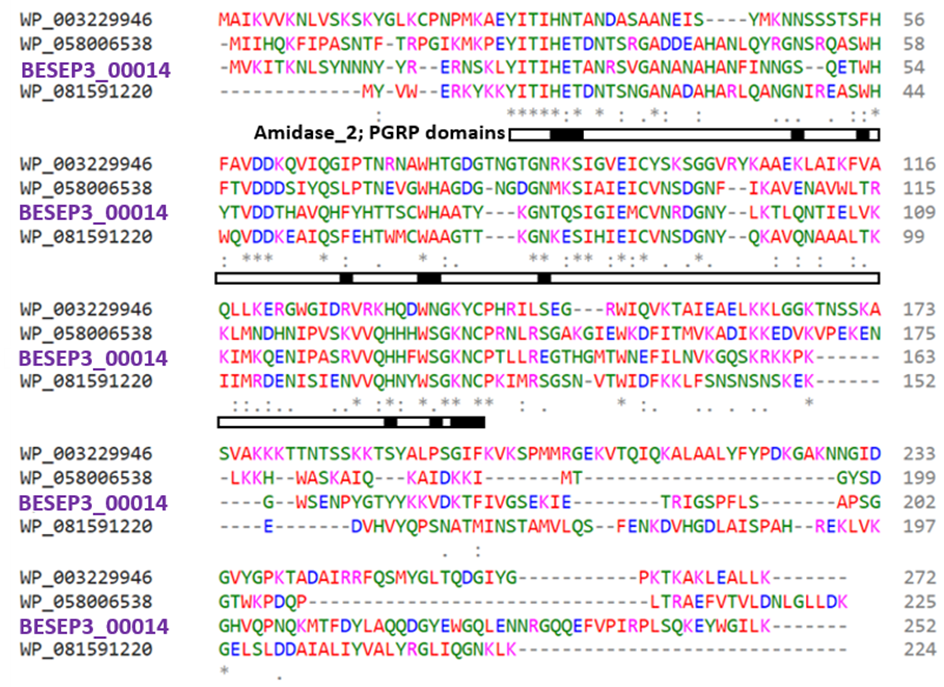
**

**Figure S1. vB_SepP_BE03 codes for a novel amidase BESEP3_00014.** BESEP3_00014 is a putative N-acetylmuramoyl-L-alanine amidase based on Clustal Omega alignment with known amidases. Structural domains are depicted by black bordered boxes. Solid black regions are putative catalytic residues.


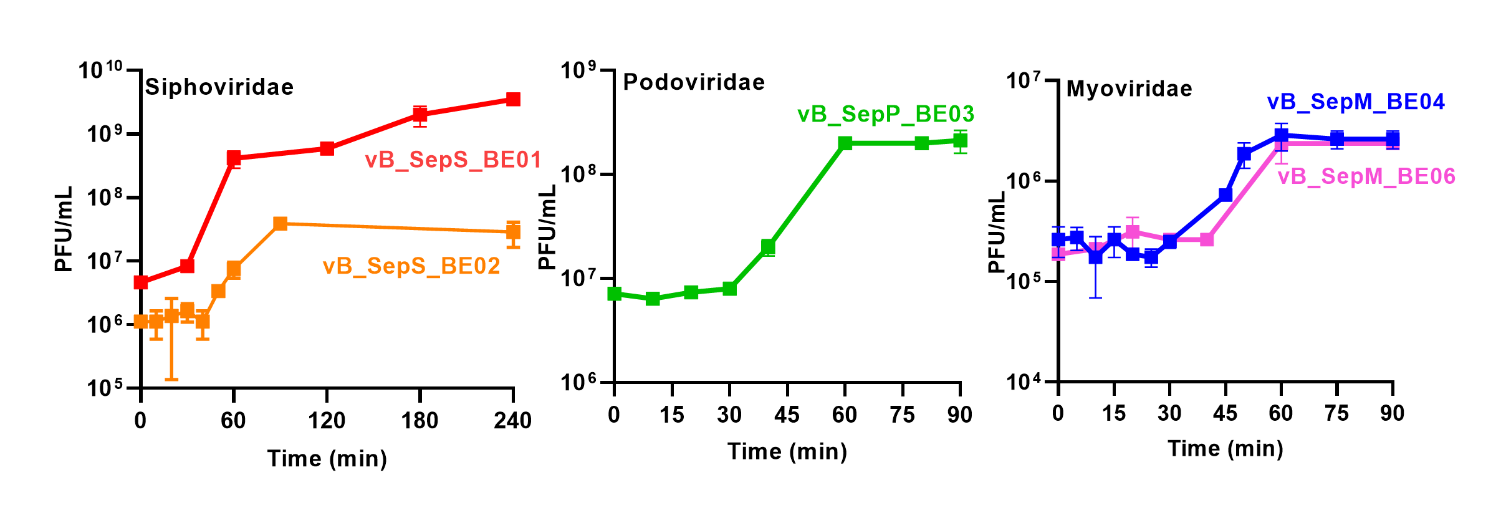
**Figure S2. One step growth curves of unique phages identified in this study.** For each experiment, the initial concentration of bacteria was ~1x10^8^ colony forming units/ml. PFU, plaque forming units.

**
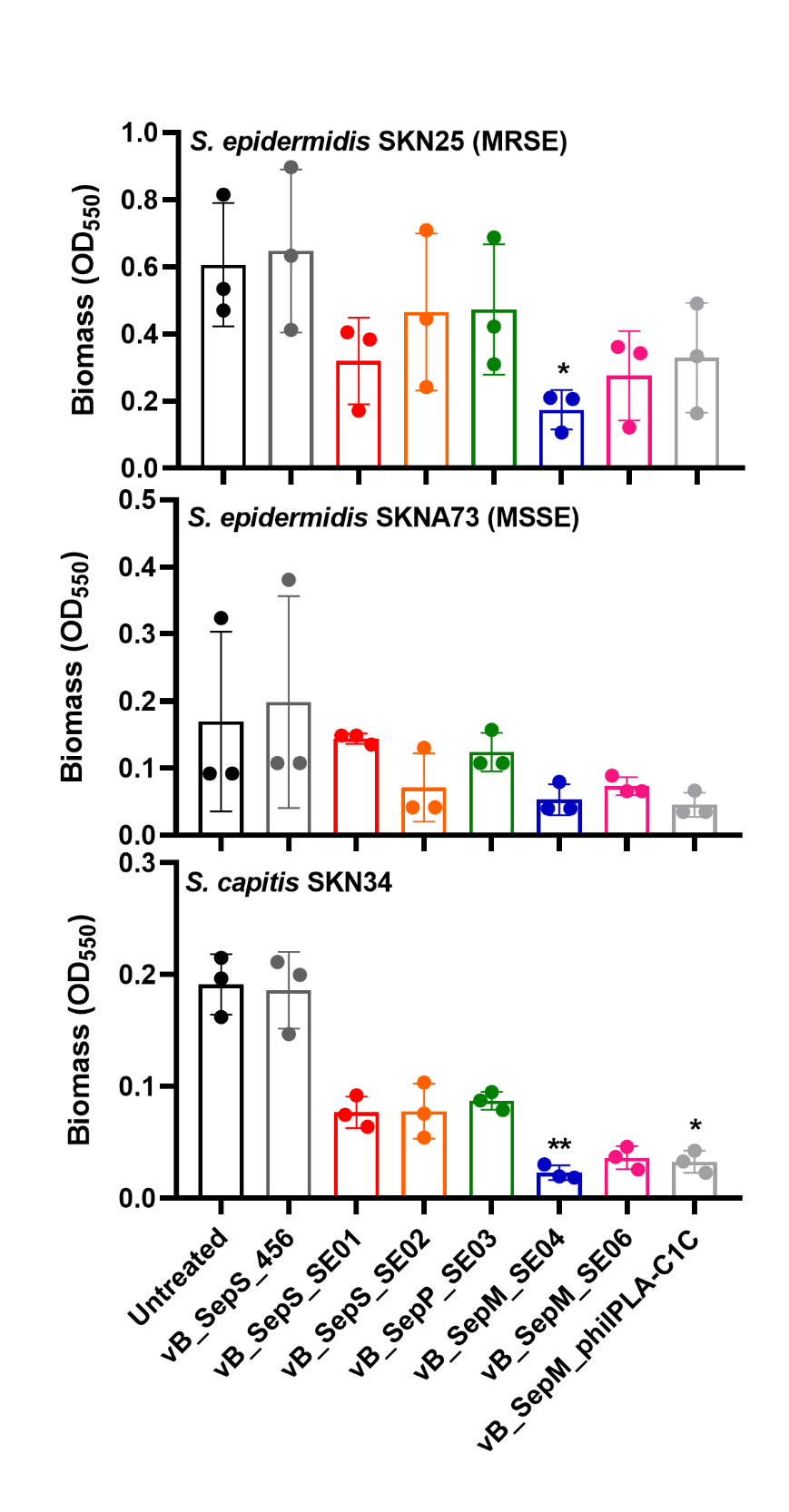
**

**Figure S3. Treatment of staphylococcal biofilms formed in 96-well plates.** Biofilms were grown for 24 hours in vitro then treated with phages (~1x10^8^ plaque forming units/ml) for 4 hours. The untreated control was treated with SM-buffer. Biomass was inferred by optical density (OD) at 550nm following crystal violet staining. Statistical differences were determined using Kruskal-Wallis tests with multiple comparisons corrected for using Dunn’s method. *p<0.05, **p<0.01.
