# Supplementary material for: Isolation and characterization of bacteriophages from the human skin microbiome that infect Staphylococcus epidermidis
Source: FEMS Microbes. 2021 Mar 30;2:xtab003. doi: 10.1093/femsmc/xtab003 (PMC10117716; doi:10.1093/femsmc/xtab003)
Supplement: xtab003_Supplemental_Files [file xtab003_supplemental_files.zip › Peer_review_comments_for_FEMSMC-2021-006.docx]

**Peer review comments for FEMSMC-2021-006**

Referee 1: Prefers to remain anonymous

The manuscript submitted by Valente and colleagues presents the isolation and characterization of Staphylococcus epidermidis phages for a future application in phage therapy. Bacteriophages have been classified by morphology and genomic characterization. The host range for each phage was determined. Finally, the ability to destroy biofilms was confirmed for one phage.

General comments: This is an interesting work as previously described S. epidermidis phages are scarce or poorly characterized and, their application on phage therapy is unexploited. The manuscript is well written and the experiments are clearly explained and discussed. However, there are some important data missing here. The biological characterization of phages (one-step growth curve, BIMs frequency, presence of virulence genes). I also suggest improving the discussion.

Minor comments:

Line 189: “putative lysogens of S. epidermidis strains SKN21, SKNA21 and SKNA60 were tested for the presence of integrated vB_SepS_BE01”. Why these strains and no other were used for lysogeny determination?.

Line 195: “For vB_SepS_BE02, no putative lysogens were identified. Instead, the host S. epidermidis strain used to propagate vB_SepS_BE02 was tested for integration using primers specific to amplify a 937bp fragment of the phage”. Please clarify, it is not clear what strains were used in each case.

Line 245. “Phage containing plaques were identified on lawns of human-host specific..”. I understood that each phage sample was only plaqued in the strain obtained from the same person. Is this correct?. Therefore, the probability to find specific phages for that strain is high but also, you can find prophages (temperate phages) released from this strain, although these will be not detected due to the superinfection immunity.

Line 251: “In total, 7 phages that could be stably propagated..” Was each phage propagated in its strain?

Figure 4. I suggest enlarging the size of the genomes, in order to properly identify each part.

Line 254. Some of the new phages have lysogeny-related genes, therefore, this title is confusing.

Line 306. It is quite difficult to define narrow and wide host range, but some of the new identified phages infect a quite significant number of strains, therefore, they can be consider as wide.

I suggest to include more information about protein homology in all the phage genomes (it can be as supplementary material) and most important, to study the presence of undesirable genes (virulence, antibiotic resistance).

Regarding phage therapy, you should also consider the bacteriophage insensitive mutants (BIMs) frequency, which would be determined for each phage. The selection of bacteria not sensitive to the phage would be a problem for eradication of infectious diseases using phages.

Referee 2: Prefers to remain anonymous

This research by Valente et al., on “Isolation and characterisation of bacteriophages from the human skin microbiome that infect Staphylococcus epidermidis” is very interesting and covers the important topic on bacteriophages. The research work was very well planned and executed. The manuscript was well written and suitable for publications.

Comments:

1. As the authors stated, it is surprising to see 24/26 individuals (11 with phages) to be positive for the presence of S. epidermidis in forehead. Is it anything to do with location or population or nature of sampling? Can you discuss?

2. Why Siphoviruses cannot be used for therapy? (Line no:364). This has to be clarified in the sentence.

3. What are the applications of these phages, as they have narrow host-range? Discuss in brief.

Author response to referees’ comments:

We thank the editor and reviewers for their helpful suggestions. Below, we have included a point-by-point response to each comment. Line numbers refer to those in the track changed documents. Changes in that document are indicated by red text.

Handling Editor Comments to Authors:

Both reviewers thought the work was interesting and novel and have suggested minor revisions to the manuscript and perhaps improving the discussion. The authors need to address all the concerns of the reviewers, especially those suggesting that there is some important data missing relating to the biological characterization of phages (one-step growth curve, BIMs frequency, presence of virulence genes). The presence or absence of virulence genes or a strong rationale for why not testing for these needs to be provided.

RESPONSE: We thank the editor for handling our manuscript. As requested, we have now included one-step growth curve data, BIM frequencies, and an analysis of the presence/absence of virulence and resistance genes, and updated the text where appropriate.

Reviewer #1

The manuscript submitted by Valente and colleagues presents the isolation and characterization of Staphylococcus epidermidis phages for a future application in phage therapy. Bacteriophages have been classified by morphology and genomic characterization. The host range for each phage was determined. Finally, the ability to destroy biofilms was confirmed for one phage.

General comments:

This is an interesting work as previously described S. epidermidis phages are scarce or poorly characterized and, their application on phage therapy is unexploited. The manuscript is well written and the experiments are clearly explained and discussed.

RESPONSE: We appreciate the positive response of reviewer #1.

- However, there are some important data missing here. The biological characterization of phages (one-step growth curve, BIMs frequency, presence of virulence genes). I also suggest improving the discussion.

RESPONSE: One-step growth curves were included (Figure S2, table S2, lines 332-333), and we have addressed each of the other deficiencies below.

Minor comments:

- Line 189: “putative lysogens of S. epidermidis strains SKN21, SKNA21 and SKNA60 were tested for the presence of integrated vB_SepS_BE01”. Why these strains and no other were used for lysogeny determination?

RESPONSE: All strains listed in Supplemental table 1 were exposed to vB_SepS_BE01 in the spot test assay, only putative lysogens formed on SKN21, SKNA21 and SKN60. Additional clarification has been added (lines 191-193).

- Line 195: “For vB_SepS_BE02, no putative lysogens were identified. Instead, the host S. epidermidis strain used to propagate vB_SepS_BE02 was tested for integration using primers specific to amplify a 937bp fragment of the phage”. Please clarify, it is not clear what strains were used in each case.

RESPONSE: We used the host strain of vB_SepS_BE02, which is S. epidermidis strain SKNA40. Additional details have been added for clarification (lines 200-203).

- Line 245. “Phage containing plaques were identified on lawns of human-host specific.”. I understood that each phage sample was only plaqued in the strain obtained from the same person. Is this correct? Therefore, the probability to find specific phages for that strain is high but also, you can find prophages (temperate phages) released from this strain, although these will be not detected due to the superinfection immunity.

RESPONSE: The comment is correct.

- Line 251: “In total, 7 phages that could be stably propagated.” Was each phage propagated in its strain?

RESPONSE: Yes, in the first instance, each phage was propagated in its own host strain. Afterwards, in order to compare phages during the host range and the biofilm disruption assays, they all were propagated in S. epidermidis F12, with the exception for vB_SepP_BE03, which was propagated in SKNA73. This has been clarified in the text (lines 178-179).

- Figure 4. I suggest enlarging the size of the genomes, in order to properly identify each part.

RESPONSE: The figure has been modified so that the genomes are larger.

- Line 254. Some of the new phages have lysogeny-related genes, therefore, this title is confusing.

RESPONSE: Titles have been changed (lines 259 and 276).

- Line 306. It is quite difficult to define narrow and wide host range, but some of the new identified phages infect a quite significant number of strains, therefore, they can be considered as wide.

RESPONSE: We agree that it is difficult to assign narrow or wide host range classification to phages, thus we modified the title to “S. epidermidis phages can infect diverse staphylococcal isolates including methicillin-resistant examples” (line 330-331).

- I suggest to include more information about protein homology in all the phage genomes (it can be as supplementary material) and most important, to study the presence of undesirable genes (virulence, antibiotic resistance).

RESPONSE: Protein homology has been inferred based on the genome sequence of each phage using both prokka and PHASTER. This information is submitted to DDBJ/ENA/GenBank and will be made available upon publication. In response to the reviewer comment, we looked for virulence and antibiotic resistance genes using the Comprehensive Antibiotic Resistance Database (CARD) (https://card.mcmaster.ca) and the VirulenceFinder 2.0 database (https://cge.cbs.dtu.dk/services/VirulenceFinder/). This information has been added in the methods (line 168-171), result section (lines 326-328) and the appropriate references were included.

- Regarding phage therapy, you should also consider the bacteriophage insensitive mutants (BIMs) frequency, which would be determined for each phage. The selection of bacteria not sensitive to the phage would be a problem for eradication of infectious diseases using phages.

RESPONSE: BIM frequencies were determined and results are now included (lines 356-358; Table S2).

Reviewer #2

This research by Valente et al., on “Isolation and characterisation of bacteriophages from the human skin microbiome that infect Staphylococcus epidermidis” is very interesting and covers the important topic on bacteriophages. The research work was very well planned and executed. The manuscript was well written and suitable for publications.

RESPONSE: We appreciate the positive response of reviewer #2.

1. As the authors stated, it is surprising to see 24/26 individuals (11 with phages) to be positive for the presence of S. epidermidis in forehead. Is it anything to do with location or population or nature of sampling? Can you discuss?

RESPONSE: Based on a previous study (Kloos WE & Musselwhite MS (1975) Distribution and persistence of Staphylococcus and Micrococcus species and other aerobic bacteria on human skin. Applied microbiology 30: 381-385) we did expect S. epidermidis to be frequently found on the forehead which is why we chose it for the purpose of looking for suitable hosts for phage propagation.

2. Why Siphoviruses cannot be used for therapy? (Line no:364). This has to be clarified in the sentence.

RESPONSE: The explanation is provided in the subsequent sentence.

“Temperate phages can integrate foreign genetic material into the genome of the host strain, which may inadvertently equip the infective bacteria with genes that contribute to antibiotic resistance or enhanced pathogenicity” (line 392-395).

3. What are the applications of these phages, as they have narrow host-range? Discuss in brief.

RESPONSE: We think that the application of such phages with a narrow host-range might be in the scope of a “sur-mesure” approach leading to a patient tailored treatment, as discussed in lines 441-444. Due to their often chronic and slowly progressing nature, this approach might be especially appropriate for infections elicited by S. epidermidis.

In light of these improvements, we believe the manuscript is now ready for publication in FEMS Microbes.
